# Intriguing Insights From 100 Consecutive Colorectal Cancer Cases in Mid-Kerala: Sparse BRAF Gene Mutations and Mismatch Repair Deficiency (MMR-D)

**DOI:** 10.7759/cureus.64133

**Published:** 2024-07-09

**Authors:** Nisha M Das, Ravindran Chirukandath, Veeshma P B, Sumin V Sulaiman, Aswathi Sooraj, Soorya Gayathry P, Jeffy John, Dona Maria Joseph, Chitra Menon

**Affiliations:** 1 Pathology, Government Medical College & Hospital, Thrissur, IND; 2 General Surgery, Government Medical College & Hospital, Thrissur, IND; 3 Pathology, Medical Trust Hospital, Cochin, IND

**Keywords:** molecular behavior, msi, braf, oncogenesis, colorectal cancer

## Abstract

Colorectal cancer (CRC) is among the most prevalent types of cancer globally. It is well established that the development of CRC primarily results from the sequential activation of oncogenes and the simultaneous inactivation of tumor suppressor genes. It has also been noted that after the initial oncogenic mutation, many subpopulations with different mutational profiles are created, causing heterogeneity among the tumors.

This retrospective study analyzed 100 patients diagnosed with CRC through colectomy over an eighteen-month period at a tertiary referral center in mid-Kerala, India. Pathology records and histological slides were reviewed by two pathologists, and clinicopathological data were collected from pathology reports. Immunohistochemical analysis for BRAF mutation and possible microsatellite instability (MSI) (by mismatch repair (MMR) protein study) was conducted on tumor tissue blocks sent to an external center due to the lack of an automated platform at the hospital. The study utilized Roche's Benchmark XT platform for BRAF analysis and assessed MMR protein expression using antibodies for MLH1, MSH2, MSH6, and PMS2.

The mean age of patients was 58.36 years, with a male predominance (58.0%). Most tumors were classified as T3 (71.0%, n-71) and T2/T4a (14.0% each, n-14), while nodal involvement included N0 (35.0%, n-35), N1 (26.0%, n-26), N2 (19.0%, n-19), and NX (20.0%, n-20). Histological examination revealed predominantly well-differentiated tumors (78.0%, n-78), with lymphatic invasion noted in 41.0% (n-41) and vascular invasion in 5.0% (n-5) of cases. Left-sided tumors predominated (33.0%, n-33), followed by rectal carcinoma (37.0%, n-37), and right-sided colon cancers (30.0%, n-30).

Genetic profiling showed sparse BRAF mutations (1.0%, n-1) and MSI (1.0%, n-1), with some cases exhibiting loss of MMR proteins (MLH1, PMS2, MSH2, and MSH6) by immunohistochemistry (IHC). The study highlights the rarity of BRAF mutations in this cohort and emphasizes the diverse pathological and molecular characteristics observed.

The discussion focuses on the implications of these findings, suggesting that CRC in this population exhibits unique clinicopathological features potentially influenced by factors beyond genetic mutations. Further multicentric studies are warranted to comprehensively explore these factors and refine risk stratification and treatment strategies for CRC patients in similar demographics.

## Introduction

Colorectal cancer (CRC) ranks among the most prevalent types of cancer globally. It’s widely known that sequential activation of oncogenes and concomitant inactivation of tumor suppressor genes is the principle process behind the development of CRC [1]. It has also been noted that after the initial oncogenic mutation, many subpopulations with different mutational profiles get created, causing heterogeneity among the tumors [2].

BRAF gene mutation is an example of such a heterogenic event in colorectal carcinogenesis and is seen in about 10% of the CRC cases in Western literature [3]. The BRAF mutation confers poor prognosis and resistance to anti-epidermal growth factor receptor (anti-EGFR) therapy. Treatment of CRC with BRAF mutations has changed rapidly in the last few years, with new combinations and targeted therapies. 

Very few studies have evaluated the prevalence and association of BRAF mutations with clinicopathological features in the Indian population. BRAF mutation frequency ranges from 2% to 7% in Indian CRC patients [4-6]. Although the BRAF mutation does not seem to be prevalent in the Indian population, it was noted to be associated with advanced stages [6]. Understanding the frequency of somatic mutations in a particular population is important in deciding the targeted therapy. 

Another putative biomarker with clinical and therapeutic potential is the presence of microsatellite instability (MSI) detected by polymerase chain reaction (PCR) or its equivalent by looking for mismatch repair (MMR) proteins by immunohistochemistry (IHC). Microsatellites are multiple repeats of 1-5 base pairs occurring at microsatellite loci, widely dispersed throughout the genome. MSI has been frequently observed in several types of cancer, including endometrial and colorectal carcinomas [7].

We retrospectively analyzed the presence of BRAF and MMR aberrations and their correlation with the clinicopathological factors in 100 colorectal cancers of the 100 cases analyzed for BRAF and MMR.

## Materials and methods

The study was conducted at Government Medical College, Thrissur, Kerala, India. We conducted a retrospective study in 100 patients diagnosed with CRC through colectomy during the period of one and a half years. Pathology records and histological slides with a diagnosis of CRC were reviewed by two pathologists. Clinicopathological information and patient history were collected from pathology reports. 

Immunohistochemical analysis for BRAF mutation and MMR status was performed on blocks of tumor tissue collected during the study period. As the standardized, automated platform for immunohistochemistry (IHC) is not available in the hospital, the blocks were sent for IHC analysis to another center. The approved system for IHC studies on BRAF is Roche (Ventana) Medical System's Benchmark XT platform and Opti View DAB IHC Detection Kit. Samples were tested using the anti-BRAF V600E (VE1) antibody on the Benchmark XT platform, and protein expression was detected using the Opti View DAB IHC Detection Kit.

MMR protein expression by IHC is a widely accepted test that identifies an affected gene by detecting the loss of protein products. The MMR protein expression profile was assessed using the Roche platform using antibodies to look for specific mismatch gene products, MLH1, MSH2, MSH6, and PMS2. Each of the markers will be analyzed for their nuclear expression (normal/abnormal). 

The intensity of BRAF expression in tumor cells was assessed using a scale ranging from 0 to 3. Strong cytoplasmic staining received a score of 3, medium cytoplasmic staining was scored as 2, weak cytoplasmic staining as 1, and absence of staining as 0. Scores of 1 to 3 indicated positive staining, while scores below 1 were considered negative staining.

The results were categorized based on the immunohistochemical profile of each case. The pathological and molecular characteristics of the tumor tissue, along with the tumor stage, were established based on the American Joint Committee on Cancer (AJCC) tumor/node/metastasis criteria [8]. 

Different clinicopathological parameters, including age, sex, location, size, differentiation, and histology, were analyzed and correlated with the IHC profile of BRAF. 

The data on these mutations and their correlation with the different clinicopathological features in the Indian cohort is inadequate.

## Results

The study was conducted on 100 consecutive colorectal cases operated on for 18 months at a tertiary referral center in mid-Kerala, India. 

The mean age in our study was 58.36±11.49 years, and out of 100 participants, 58 (58.0%) were male and 42 (42.0%) were female. 

Most of the patients underwent radical colectomy and, hence, constituted the majority. In our study, the prevalence of each stage was found to be as follows: T3 (71.0%), T2 (14.0%), and T4a (14.0%). Early tumors T1 (1.0%) were sparse and constituted only 1% of the total resected specimens. The majority of resected tumors (35%) were lymph node-negative specimens, which may be due to unstandardized resections. The rest was made up of N1 (26.0%), NX (20.0%), and N2 (19.0%) (Table 1).

**Table 1:** Distribution of TNM stage TNM: tumor, node, metastasis

TNM stage	Frequency (n=100)	Percentage
T1	1	1%
T2	14	14%
T3	71	71%
T4a	14	14%
NX	20	20%
N0	35	35%
N1	26	26%
N2	19	19%
MX	100	100%

The tumors were analyzed for the degree of differentiation and lymphovascular invasion (LVI), wherein 41% of tumors exhibited lymphatic permeation but vascular emboli were seen only in 5%. Tumor differentiation also had a significant predilection for low-grade tumors, with 78% of the tumors exhibiting good differentiation and 9% exhibiting poor differentiation. These factors point to the fact that these tumors were less aggressive on the basis of their pathological behavior (Table 2, 3).

**Table 2:** Distribution of pathological diagnosis

Pathological diagnosis	Frequency (n=100)	Percentage
Large vessel invasion	5	5.0%
Lymphatic invasion	41	41.0%

**Table 3:** Distribution of histological grading

Histological grade	Frequency	Percentage
Well differentiated	78	78.0%
Moderate	13	13.0%
Poor	9	9.0%

Location-wise, most of the tumors were left-sided colonic (33%). Rectal carcinoma constituted 37.0%, and right-sided tumors contributed to 30% of the tumors.

**Figure 1:**
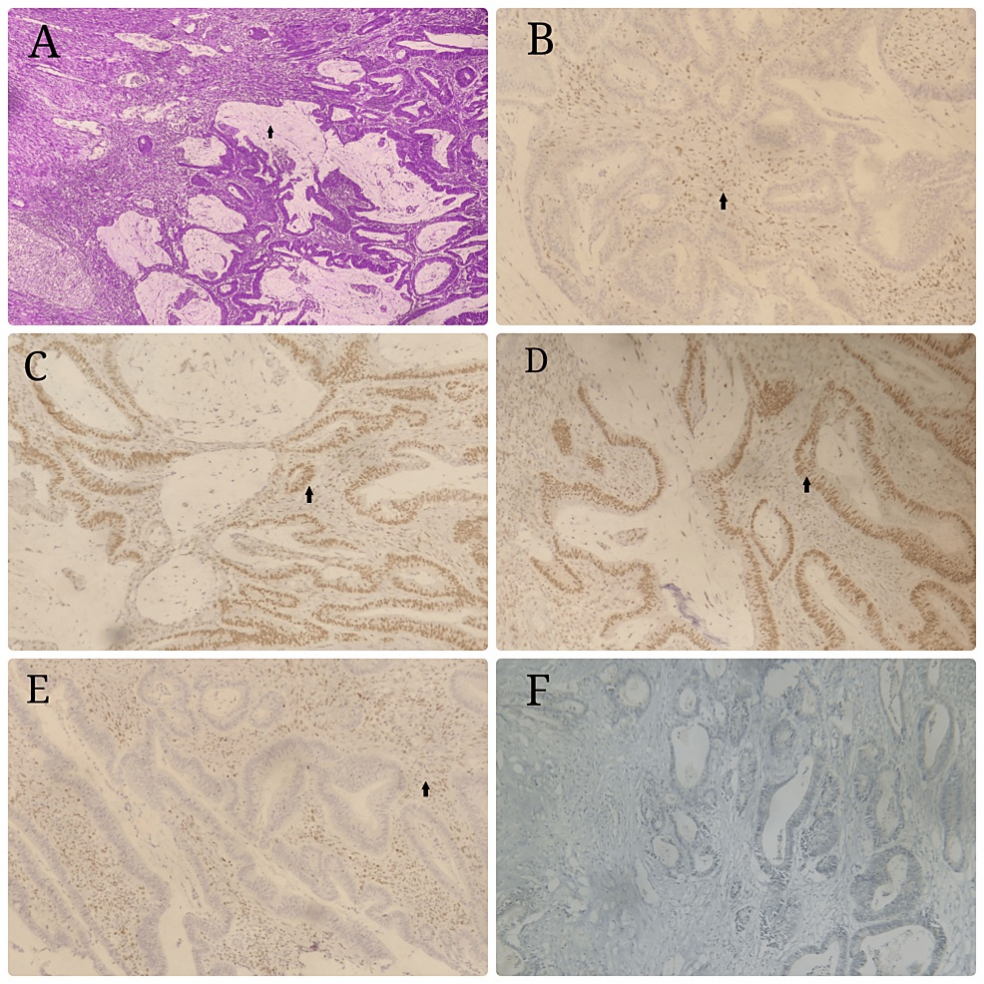
Genetic mutation and histopathology A: colonic carcinoma with peritumoral lymphocytic reaction; B: loss of nuclear expression of MSH 6; C: intact nuclear expression of PMS2; D: nuclear expression of MLH 1; E: loss of expression of MSH 2; F: BRAF negative

Loss of MSH2 and MSH6 protein expression with normal MLH1, PMS2, and BRAF was seen in three patients. There were two cases with loss of MLH1 and PMS with no aberrations of MSH2, MSH6, or BRAF. There was only one case of positive BRAF with normal MLH, MSH, and PMS status (Figure 1). There is no significant presence of genetic mutations, with just 6% of MSI mutations and BRAF mutations occurring in just 1% (Table 4).

**Table 4:** Distribution of genetic profile

Genetic profile	Frequency	Percentage
BRAF MUTATION	1	1.0%
MMR IHC	1	1.0%
MLH 1	1	1.0%
MSH2	1	1.0%
PMS 2	1	1.0%
MSH 6	1	1.0%

## Discussion

Colorectal cancer ranks as the third most diagnosed cancer globally, with approximately 1.4 million new cases each year. Tumors in the colorectal region that exhibit BRAF mutations and microsatellite stability (MSS) are linked to unfavorable outcomes in patients [9]. Therefore, combined testing for microsatellite instability high (MSI-H) and BRAF mutations could potentially aid in risk assessment, especially for patients diagnosed with stage II tumors [10].

Carcinogenesis in CRC can occur through multiple pathways. Unlike the classic adenoma-carcinoma sequence, where APC inactivation is the initiating event [11], the serrated pathway is driven by BRAF mutations, with serrated polyps serving as the precursor lesions [12].

Serrated tumors typically do not exhibit chromosomal instability but often show extensive DNA methylation of CpG islands. This methylation can occur in the MLH1 promoter (a gene involved in the mismatch repair system), leading to a 'sporadic' microsatellite instability (MSI) phenotype. [13]. In 60% of sporadic MSI CRC cases, there is an association with BRAF mutations. However, BRAF mutations are not found in Lynch syndrome, where microsatellite instability arises through a different mechanism involving germline mutations in mismatch repair genes.

BRAF mutations are more common in tumors of the proximal colon and are associated with unique pathological features, including poorer differentiation, mucinous histology, MSI, and larger primary tumors [14]. The metastatic pattern also differs from BRAF wild-type (BRAF-WT) tumors, showing a higher incidence of peritoneal metastasis and fewer liver and lung metastases. 

BRAF negativity in this series points to the fact that colorectal cancer etiology and behavior are influenced by other factors. The various factors that emerged from this study pointed out that the majority of the tumors studied were early-stage, left-sided, well-differentiated tumors with a low nodal load and fewer lymph vascular emboli. 

The mitogen-activated protein kinase (MAPK) cascade is pivotal in tumor cell proliferation and survival. Increasing evidence indicates that mutations in the BRAF oncogene are not only tied to a poorer prognosis but also correlate with reduced efficacy of anti-epidermal growth factor receptor antibodies in treating metastatic colorectal cancer (mCRC) [15].

Importantly, the study highlights that colorectal cancer's etiology in our population is influenced by a broader spectrum of factors beyond genetic mutations. The predominance of early-stage, left-sided tumors with well-differentiated histology, low nodal load, and minimal lymph vascular emboli suggests alternative pathogenic mechanisms. This finding calls for further multi-centric, structured analyses to comprehensively evaluate these variables and their contributions to CRC development and progression [16].

The study points to the fact that colorectal cancer in our population has diverse etiologies and factors other than genetic mutations. The factor of divergent causation needs to be further analyzed by a multi-centric analysis with a more structured approach looking at various variables. 

Limitations of the study

This study has several limitations, including its retrospective design, small sample size, and single-center data, which limit the generalizability and causality of the findings. The lack of an automated IHC platform requires sending samples to an external center, potentially introducing variability, and the focus on a limited set of genetic markers may overlook other relevant genetic alterations. Additionally, the absence of long-term follow-up data might affect the assessment of prognostic implications and pathological staging.

## Conclusions

This research underscores the complexity and heterogeneity of colorectal cancer (CRC) etiology and progression, emphasizing the role of genetic and epigenetic alterations. The identification of BRAF mutations and microsatellite instability (MSI) as markers associated with adverse patient outcomes highlights the importance of integrating molecular diagnostics into risk assessment, particularly for early-stage CRC patients. Insights into tumor biology suggest that BRAF mutations contribute to more aggressive disease phenotypes and may influence therapeutic responses, particularly regarding anti-epidermal growth factor receptor antibody treatments in metastatic CRC.

In conclusion, while genetic markers such as BRAF mutations and MMR/microsatellite status provide critical insights into CRC prognosis and therapeutic strategies, a more nuanced understanding of the diverse etiological factors is essential. Such efforts could pave the way for more personalized and effective diagnostic, prognostic, and therapeutic strategies for managing colorectal cancer.
